# Risk Factors for Postoperative Infections in Cardiac Surgery Patients: A Retrospective Study

**DOI:** 10.7759/cureus.43614

**Published:** 2023-08-17

**Authors:** Abdulkarim Abukhodair, Mohammed S Alqarni, Abdulmalek Alzahrani, Ziad M Bukhari, Ammar Kadi, Faisal M Baabbad, Abdullah Algarni, Sahal Jamalallail, Mohammed Almohammadi, Sean R Bennett

**Affiliations:** 1 Medicine, King Saud Bin Abdulaziz University for Health Sciences, Jeddah, SAU; 2 Internal Medicine, King Abdullah International Medical Research Center, Jeddah, SAU; 3 Internal Medicine, National Guard Hospital, King Abdulaziz Medical City, Jeddah, SAU; 4 Radiology, King Faisal Specialist Hospital and Research Center, Jeddah, SAU; 5 Medicine, King Abdullah International Medical Research Center, Jeddah, SAU; 6 College of Medicine, King Saud Bin Abdulaziz University for Health Sciences, Jeddah, SAU; 7 Medicine, King Saud Bin Abdulaziz University for Health Sciences College of Medicine, Jeddah, SAU; 8 Emergency Medicine, Ministry of National Guard Health Affairs, Riyadh, SAU; 9 Pathology and Laboratory Medicine, National Guard Hospital, King Abdulaziz Medical City, Jeddah, SAU; 10 Hematopathology and Transfusion Medicine, National Guard Hospital, King Abdulaziz Medical City, Jeddah, SAU; 11 Anesthesiology/Cardiac and Intensive Care, University Hospital Southampton NHS Foundation Trust, Southampton, GBR

**Keywords:** heart failure, copd, hypertension, infection, cardiac surgery, blood transfusion

## Abstract

Background and objective

Cardiac surgery is one of the most common surgical procedures globally; its incidence has been on the rise due to the faster pace of population aging thanks to technological and epidemiological advances. Patients who undergo cardiac surgeries may face various postoperative complications that might affect their survival, and one of these major complications is infection. Nosocomial pneumonia, surgical site infection (SSI), mediastinitis, bacteremia, and sepsis are common infections encountered after surgeries. In this study, we aimed to determine the common risk factors related to postoperative infections at the King Faisal Cardiac Center from January 2014 to September 2020.

Materials and methods

Records from 364 patients who underwent cardiac surgery and were aged above 18 years were assessed for postoperative infections in this retrospective cohort study. Patients who were immunosuppressed or had active systemic infections were excluded. Consent was waived by the Institutional Review Board. All procedures were performed at the King Faisal Cardiac Center, National Guard Hospital, Jeddah.

Results

Of the total 364 patients, 105 were women and 259 were men. The mean age of the cohort was 59 years (SD = 13) and the mean BMI was 29.1 kg/m^2^ (SD = 5.3). The study population showed a high prevalence of cardiac risk factors and diseases: diabetes (n = 244, 67%), hypertension (n = 230, 63%), dyslipidemia (n = 144, 40%), smoking (n = 80, 22%), heart failure (n = 41, 11%), and chronic obstructive pulmonary disease (n = 6, 1.6%). The overall rate of postoperative infection was 32.7% (n = 120), and 17 (14%) of these infected patients underwent reoperations for infection.

Conclusion

Based on a thorough analysis of 364 patients undergoing various cardiac surgical procedures, including a multivariate analysis accounting for preoperative factors, there was a significant association between postoperative infections and hypertension, diabetes, increased preoperative activated partial thromboplastin time, and elevated HbA1c.

## Introduction

Cardiac surgery is one of the most common surgical interventions worldwide. Its incidence has increased with the increase in the pace of population aging owing to technological and epidemiological advances. Coronary artery disease (CAD) and valvular disorders are among the indications for performing invasive cardiothoracic surgery. CAD has been increasing globally over the last several decades due to the increase in life expectancy. Coronary artery bypass graft (CABG) surgery is a major procedure performed to treat severe multi-vessel diseases [1,2]. Also, another indication for the invasive cardiothoracic procedure is valvular disease, where medical management does not lead to symptom control and when patients are not endovascular candidates. These operations could put the patients at a huge risk of postoperative complications as described in various published studies [3,4].

Patients may experience several postoperative complications following cardiac surgeries, which can impact their survival. One of these major complications is infection [5]. Nosocomial pneumonia, surgical site infection (SSI), mediastinitis, bacteremia, and sepsis are common infections after surgeries [6]. The most commonly occurring infections are pneumonia, Clostridioides difficile colitis, and bloodstream infections. SSIs are generally less common [7]. These infections are associated with many risk factors that have been widely studied. Some of these risks are linked to the patient’s demographics and health conditions such as age, gender, obesity, and diabetes [8-10]. On the other hand, some risk factors are linked to the procedures and hospital management-related factors, such as operation types, cardiopulmonary bypass (CPB), blood transfusions, and antibiotic prophylaxis [7,11,12,13]. The identification of these factors is critical to reduce postoperative infections and to establish a special protocol for managing high-risk patients. At the King Faisal Cardiac Center, the rate of postoperative infection has been reported to be 32%, which is extremely high [14]. In light of this, the aim of this study was to determine the common risk factor related to postoperative infections among patients at the King Faisal Cardiac Center from January 2014 to September 2020.

## Materials and methods

Records from 364 patients who underwent cardiac surgery and were above 18 years of age were assessed for postoperative infection in this retrospective cohort study. Patients who were immunosuppressed or had active systemic infections were excluded. Consent was waived by the Institutional Review Board. All procedures were performed at the King Faisal Cardiac Center, National Guard Hospital, Jeddah, between January 2014 and September 2020. Data collection was done through the BESTCare system at the medical records department of the National Guard Hospital, Jeddah. The authors reviewed the medical records in the last quarter of 2020. The study was in accordance with the tenets of the Declaration of Helsinki and was approved by the Institutional Review Board (IRB) at King Abdullah International Medical Research Center (KAIMARC) with the IRB reference number IRBC/2065/19.

For the purpose of this study, postoperative infection was defined as infections occurring six weeks after the surgery. The types of infections looked for were SSI of the chest, SSI of the leg, pneumonia, bloodstream infection, urinary tract infection (UTI), Clostridioides difficile infection, and endocarditis. SSIs were defined as purulent discharge from the wound; in addition, all infections were confirmed by swab culture. Other secondary outcomes included mortality, length of stay, and reopening of wound sites due to infection. Postoperative mortality was defined as an in-hospital death. The prophylaxis protocol at the center involves the administration of 2 gm cefazolin one-hour pre-incision, administered every four hours in the operating room, which is increased to 3 gm if the patient weight is >120 kg, and continued eight hourly postop in the ICU until it drains out. Since May 2019, gentamicin has been added at 5 mg/kg preoperatively and one further dose after 24 hours. If creatinine clearance is <60 mls, the second dose is omitted.

The independent variables analyzed were as follows: basic demographics, hypertension, diabetes, dyslipidemia, heart failure, chronic obstructive pulmonary disease, history of smoking, type of operation, surgery duration, glycated hemoglobin, preoperative labs and coagulation profile, postoperative labs and coagulation profile, and transfusion status. For statistical analysis, the chi-square test was used for categorical variables, and the Wilcoxon rank sum test was used for continuous variables. Univariate and multivariate regression analyses were used to identify the association between independent factors and postoperative infection. A p-value of less than 0.05 was considered statistically significant. All statistical analyses and assessments of the model’s performance were conducted using the R software, version 3.3.0 (R Foundation for Statistical Computing, Vienna, Austria) [15].

## Results

There were 105 women and 259 men in the study. The mean age of the participants was 59 years (SD = 13) and the mean BMI was 29.1 kg/m^2^ (SD = 5.3). The population showed a high prevalence of cardiac risk factors and diseases: diabetes (n = 244, 67%), hypertension (n = 230, 63%), dyslipidemia (n = 144, 40%), smoking (n = 80, 22%), heart failure (n = 41, 11%), and chronic obstructive pulmonary disease (n = 6, 1.6%). Table 1 shows various descriptive statistics and perioperative patient parameters stratified by infection status.

**Table 1:** Characteristics of patients stratified by infection status ^1^Statistical tests performed: chi-square test of independence; Wilcoxon rank sum test; Fisher's exact test The table above shows various descriptive statistics stratified by infection status. Each variable has a p-value comparing descriptive variables across the infection variable. If the variable in the table was a categorical variable the test used was a chi-square test, and if it was continuous, a Wilcoxon-Ranked sum test was used. A p-value less than 0.05 would be considered statistically significant SD: standard deviation; COPD: chronic obstructive pulmonary disease; IQR: interquartile range; CABG: coronary artery bypass graft surgery; VR: valvular replacement; PRBC: packed red blood cells; INR: international normalized ratio; PT: prothrombin time; aPTT: activated partial thromboplastin time; HbA1c: hemoglobin A1C; LOS: length of stay

Characteristic	Overall (n = 364)	Infection status: no (n = 245)	Infection status: yes (n = 119)	P-value^1^
Gender, n (%)				0.077
Female	105 (29%)	63 (26%)	42 (35%)	
Male	259 (71%)	182 (74%)	77 (65%)	
Age, years				0.2
Median (IQR)	60 (51, 68)	60 (49, 68)	61 (54, 68)	
Mean (SD)	59 (13)	58 (14)	60 (12)	
Weight, kg				0.3
Median (IQR)	76 (67, 88)	77 (67, 89)	74 (66, 85)	
Mean (SD)	77 (15)	78 (15)	76 (13)	
Height, meters				0.019
Median (IQR)	1.63 (1.57, 1.70)	1.64 (1.58, 1.71)	1.62 (1.55, 1.69)	
Mean (SD)	1.63 (0.09)	1.64 (0.09)	1.62 (0.09)	
Body mass index, kg/m^2^				0.8
Median (IQR)	28.9 (25.3, 32.8)	28.9 (25.4, 32.4)	28.4 (25.1, 32.9)	
Mean (SD)	29.1 (5.3)	29.0 (5.3)	29.3 (5.3)	
Unknown	1	1	0	
Smoking, n (%)				0.5
No	284 (78%)	188 (77%)	96 (81%)	
Yes	80 (22%)	57 (23%)	23 (19%)	
Type 2 diabetes, n (%)				0.002
No	120 (33%)	94 (38%)	26 (22%)	
Yes	244 (67%)	151 (62%)	93 (78%)	
COPD, n (%)				>0.9
No	358 (98%)	241 (98%)	117 (98%)	
Yes	6 (1.6%)	4 (1.6%)	2 (1.7%)	
Heart failure, n (%)				<0.001
No	323 (89%)	229 (93%)	94 (79%)	
Yes	41 (11%)	16 (6.5%)	25 (21%)	
Hypertension, n (%)				0.017
No	134 (37%)	101 (41%)	33 (28%)	
Yes	230 (63%)	144 (59%)	86 (72%)	
Dyslipidemia, n (%)				>0.9
No	220 (60%)	149 (61%)	71 (60%)	
Yes	144 (40%)	96 (39%)	48 (40%)	
Antibiotic, n (%)				0.9
2 gm cefazolin	343 (94%)	230 (94%)	113 (95%)	
2 gm cefazolin + gentamicin	21 (5.8%)	15 (6.1%)	6 (5.0%)	
Operation, n (%)				0.4
CABG	239 (66%)	157 (64%)	82 (69%)	
CABG + VR	26 (7.1%)	17 (6.9%)	9 (7.6%)	
Other	20 (5.5%)	17 (6.9%)	3 (2.5%)	
VR	79 (22%)	54 (22%)	25 (21%)	
Duration of surgery, minutes				0.6
Median (IQR)	260 (225, 319)	262 (225, 325)	258 (226, 311)	
Mean (SD)	280 (116)	284 (123)	273 (102)	
Unknown	1	1	0	
Preop hemoglobin, g/dl				0.011
Median (IQR)	13.00 (11.65, 14.30)	13.20 (11.80, 14.40)	12.55 (11.20, 13.70)	
Mean (SD)	12.87 (2.03)	13.08 (1.97)	12.45 (2.10)	
Unknown	1	0	1	
Postop hemoglobin, g/dl				0.2
Median (IQR)	9.50 (8.60, 10.60)	9.60 (8.60, 10.70)	9.30 (8.50, 10.40)	
Mean (SD)	9.68 (1.57)	9.76 (1.57)	9.52 (1.57)	
Hemoglobin at discharge, g/dl				0.6
Median (IQR)	10.40 (9.60, 11.20)	10.40 (9.60, 11.40)	10.40 (9.70, 11.00)	
Mean (SD)	10.53 (1.49)	10.56 (1.58)	10.47 (1.27)	
Preop creatinine, µmol/L				0.8
Median (IQR)	78 (68, 99)	78 (68, 97)	78 (68, 102)	
Mean (SD)	101 (98)	103 (112)	98 (60)	
Postop creatinine, µmol/L				0.13
Median (IQR)	81 (68, 113)	80 (68, 110)	84 (70, 124)	
Mean (SD)	115 (115)	110 (120)	123 (104)	
HbA1c, %				<0.001
Median (IQR)	6.80 (5.80, 8.40)	6.40 (5.60, 8.00)	7.50 (6.35, 9.05)	
Mean (SD)	7.31 (2.07)	7.00 (1.87)	7.94 (2.31)	
Transfusion, n (%)				0.2
No	50 (14%)	38 (16%)	12 (10%)	
Yes	314 (86%)	207 (84%)	107 (90%)	
PRBC transfusion, n (%)				0.2
No	53 (15%)	40 (16%)	13 (11%)	
Yes	310 (85%)	205 (84%)	105 (89%)	
Unknown	1	0	1	
PRBC transfusion amount, mL/kg				0.051
Median (IQR)	2.00 (1.00, 4.00)	2.00 (1.00, 3.00)	2.00 (1.00, 4.00)	
Mean (SD)	2.76 (2.71)	2.54 (2.52)	3.22 (3.01)	
Platelet transfusion, n (%)				>0.9
No	284 (78%)	191 (78%)	93 (78%)	
Yes	80 (22%)	54 (22%)	26 (22%)	
Preop INR				0.9
Median (IQR)	1.10 (1.00, 1.10)	1.10 (1.00, 1.10)	1.10 (1.00, 1.10)	
Mean (SD)	1.10 (0.17)	1.11 (0.17)	1.10 (0.17)	
Postop INR				0.3
Median (IQR)	1.20 (1.10, 1.20)	1.20 (1.10, 1.30)	1.20 (1.10, 1.20)	
Mean (SD)	1.20 (0.24)	1.20 (0.27)	1.20 (0.17)	
Preop PT, seconds				0.4
Median (IQR)	13.00 (12.00, 14.00)	13.00 (12.00, 14.00)	13.00 (12.00, 14.00)	
Mean (SD)	13.16 (2.12)	13.16 (2.21)	13.18 (1.92)	
Postop PT, seconds				0.5
Median (IQR)	14.00 (13.00, 15.00)	14.00 (13.00, 15.00)	14.00 (13.00, 15.00)	
Mean (SD)	14.22 (2.91)	14.23 (3.24)	14.19 (2.08)	
Preop aPTT, seconds				0.019
Median (IQR)	31 (28, 36)	31 (28, 35)	32 (29, 38)	
Mean (SD)	34 (11)	33 (10)	36 (12)	
Postop aPTT, seconds				0.2
Median (IQR)	33.0 (30.0, 37.0)	32.0 (29.0, 36.0)	33.0 (30.0, 37.0)	
Mean (SD)	35.0 (14.7)	34.5 (13.3)	36.0 (17.2)	
LOS, days				<0.001
Median (IQR)	12 (9, 18)	11 (9, 15)	17 (11, 22)	
Mean (SD)	15 (11)	13 (8)	19 (14)	
Mortality, n (%)				>0.9
Alive	350 (96%)	235 (96%)	115 (97%)	
Died	14 (3.8%)	10 (4.1%)	4 (3.4%)	

The overall rate of postoperative infection was 32.7% (n = 120), and 17 (14%) of the infected patients had reoperation for infection. Seventy-six patients (20.9%) had a chest or leg SSI. The most prevalent types of infection were SSIs of the chest (59), pneumonia (24), SSIs of the leg (17), and UTIs (12). Table 2 shows the distribution of various infection types. There was an increased length of stay (OR = 1.07, CI = 1.04-1.11, p<0.001) among infected patients, while mortality was not associated with infection. Seventeen (14%) of infected patients required reopening for infection.

**Table 2:** Infection rate and types of infection C. diff: Clostridioides difficile; SSI: surgical site infection; UTI: urinary tract infection

Characteristic	Values, n (%)
Infection type	
None	244 (67%)
Pneumonia	24 (6.6%)
Bloodstream	5 (1.4%)
C. diff colitis	1 (0.3%)
SSI (chest)	57 (16%)
SSI (leg)	17 (4.7%)
Endocarditis	2 (0.5%)
UTI	12 (3.3%)
Chest and other	2 (0.5%)

On univariate logistic regression analysis, variables that were significantly associated with infection included hypertension (OR = 1.3, CI = 1.14-2.97, p = 0.013), heart failure (OR = 3.81, CI = 1.96-7.59, p<0.001), diabetes (OR = 2.23, CI = 1.36-3.74, p = 0.002), increased preoperative activated partial thromboplastin time (OR = 1.02, CI = 1.00-1.04, p = 0.036), and increased HbA1c (OR = 1.24, CI = 1.12-1.39, p<0.001). For every unit increase in red blood cells administered, there was a 9% increased risk of getting an infection (OR = 1.09, CI = 1.01-1.19, p = 0.028). However, neither incidence of transfusion nor red blood cell transfusion was associated with postoperative infection (OR = 1.58, CI = 0.83-3.18, p = 0.2) (Table 3).

**Table 3:** Univariate logistic regression with infection as the outcome Each variable was tested as to whether or not they were associated with infection. Odds ratios (OR) and confidence intervals (CI), and p-values are reported. If the OR is greater than 1, it indicates that there is an increased chance of the event occurring (infection) for that variable or variable level. If the OR is less than 1, it means that the variable or variable level was less likely to be associated with the event. A p-value less than 0.05 is considered to be significant. COPD: chronic obstructive pulmonary disease; CABG: coronary artery bypass graft surgery; VR: valvular replacement; PRBC: packed red blood cells; INR: international normalized ratio; PT: prothrombin time; aPTT: activated partial thromboplastin time; HbA1c, hemoglobin A1C; LOS: length of stay

Characteristic	N	OR	95% CI	P-value
Gender	364			
Female		—	—	
Male		0.63	0.40, 1.02	0.059
Age	364	1.01	1.00, 1.03	0.14
Weight	364	0.99	0.98, 1.01	0.4
Height	364	0.05	0.00, 0.63	0.021
Body mass index	363	1.01	0.97, 1.05	0.6
Smoking	364			
No		—	—	
Yes		0.79	0.45, 1.35	0.4
Type 2 diabetes	364			
No		—	—	
Yes		2.23	1.36, 3.74	0.002
COPD	364			
No		—	—	
Yes		1.03	0.14, 5.35	>0.9
Heart failure	364			
No		—	—	
Yes		3.81	1.96, 7.59	<0.001
Hypertension	364			
No		—	—	
Yes		1.83	1.14, 2.97	0.013
Dyslipidemia	364			
No		—	—	
Yes		1.05	0.67, 1.64	0.8
Operation	364			
CABG		—	—	
CABG + VR		1.01	0.42, 2.33	>0.9
Other		0.34	0.08, 1.04	0.09
VR		0.89	0.51, 1.52	0.7
Duration of surgery	363	1	1.00, 1.00	0.4
Preop hemoglobin	363	0.86	0.76, 0.95	0.006
Antibiotic	364			
2 gm cefazolin		—	—	
2 gm cefazolin + gentamicin		0.81	0.28, 2.06	0.7
Postop hemoglobin	364	0.91	0.78, 1.04	0.2
Hemoglobin at discharge	364	0.96	0.83, 1.12	0.6
Preop creatinine	364	1	1.00, 1.00	0.7
Postop creatinine	364	1	1.00, 1.00	0.3
HbA1c	364	1.24	1.12, 1.39	<0.001
Transfusion	364			
No		—	—	
Yes		1.64	0.84, 3.39	0.2
PRBC transfusion	363			
No		—	—	
Yes		1.58	0.83, 3.18	0.2
PRBC transfusion amount	364	1.09	1.01, 1.19	0.028
Platelet transfusion	364			
No		—	—	
Yes		0.99	0.58, 1.67	>0.9
Preop INR	364	0.96	0.25, 3.35	>0.9
Postop INR	364	0.97	0.36, 2.32	>0.9
Preop PT	364	1.01	0.90, 1.11	0.9
Postop PT	364	1	0.91, 1.07	>0.9
Preop aPTT	364	1.02	1.00, 1.04	0.036
Postop aPTT	364	1.01	0.99, 1.02	0.4
LOS	364	1.07	1.04, 1.11	<0.001

In addition, multivariate logistic regression analysis was used to assess statistically significant factors as well as blood transfusion. In this model, transfusion did not significantly affect infection rates (OR = 1.04, p = 0.6), while preoperative activated partial thromboplastin time, heart failure, and hemoglobin A1c did (Table 4).

**Table 4:** Multivariate analysis For the multivariate logistic regression, odds ratios (OR), confidence intervals (CI), and p-values were reported. In the full model (left side of the table), we examined if transfusions would have a statistically significant effect on infection rates while adjusting for gender, height, diabetes mellitus type 2, heart failure, hypertension, preop hemoglobin, preop aPTT, and HbA1c. In this model, transfusion did not have a statistically significant effect on infection rates (OR = 1.04, p =0.6). In the reduced model (right side of the table), we examined the most influential variables (4 covariates and transfusion) from the full model along with transfusion to determine if the smaller model would have different results. However, transfusion was still not statistically significant in the reduced model, (OR = 1.05, p = 0.5). aPTT: activated partial thromboplastin time; HbA1c; hemoglobin A1C

Characteristic	Full model			Reduced model		
	exp(Beta)	95% CI^1^	p-value	exp(Beta)	95% CI	P-value
Transfusion						
No	—	—		—	—	
Yes	1.03	0.90, 1.19	0.6	1.05	0.91, 1.20	0.5
Heart failure						
No	—	—		—	—	
Yes	1.36	1.17, 1.57	<0.001	1.35	1.17, 1.57	<0.001
Preop hemoglobin	0.98	0.96, 1.01	0.14	0.98	0.95, 1.00	0.038
Preop aPTT	1.01	1.00, 1.01	0.005	1.01	1.00, 1.01	0.004
HbA1c	1.04	1.01, 1.07	0.004	1.05	1.03, 1.07	<0.001
Gender						
Female	—	—				
Male	0.96	0.84, 1.10	0.6			
Height	0.71	0.36, 1.41	0.3			
Type 2 diabetes						
No	—	—				
Yes	1.04	0.91, 1.19	0.5			
Hypertension						
No	—	—				
Yes	1.05	0.94, 1.18	0.3			

In the reduced model, we examined the most influential variables from the full model along with transfusion to determine if the smaller model would have different results. However, transfusion was still not statistically significant in the reduced model (OR = 1.05, CI = 0.91-1.20, p = 0.5). Type of operation, body mass index, and duration of surgery were not associated with postoperative infection. However, females undergoing pure valvular surgery had a significant reduction in the rate of infection (OR = 0.78, CI = 0.64-0.96, p = 0.018). Finally, although not significantly associated, there was a trend of males being more likely infected than female patients (OR = 0.63, CI = 0.40-1.02, p = 0.059).

## Discussion

Determining risk factors for postoperative infection is important in our efforts to establish preventative measures. It is evident that many common risk factors all increase the likelihood of developing postoperative infection, such as hypertension (OR = 1.3, CI = 1.14-2.97, p = 0.013), heart failure (OR = 3.81, CI = 1.96-7.59, p<0.001), diabetes (OR = 2.23, CI = 1.36-3.74, p = 0.002), increased preoperative activated partial thromboplastin time (OR = 1.02, CI = 1.00-1.04, p = 0.036), and increased HbA1c (OR = 1.24, CI = 1.12-1.39, p<0.001). Furthermore, infection rates do not seem to differ largely between males and females; however, the results indicate a decrease in infection rates for women undergoing valvular surgeries rather than CABG surgeries. No other links, such as those with gender and/or types of operation, are apparent. One result that we would like to examine further is the link between blood transfusions, especially red blood cell transfusion, and the risk for postoperative infection. Our findings indicate that with every unit increase in red blood cell transfusion, the postoperative infection rate may increase by 9%. However, there is no statistically significant link between the incidence of transfusion and the incidence of postoperative infection. The absence of such a link is likely due to the liberal transfusion protocol at this center, where more than 85% of patients received a red blood cell transfusion; hence, the sample of patients who have not received transfusions is too small to provide any statistically significant data. However, significant links between the incidence of infection and an increase in red blood cell transfusions have been established in other papers [7,16,17].

Preventative measures can be taken both before and after surgery. This includes the monitoring of blood pressure, HbA1C, weight, and other medications being used. Major concerns including hypertension and obesity were prominent in the sample with 63% of patients exhibiting hypertension, and both mean and median BMI values being almost 29. Losing weight and compliance with diet guidelines such as Dietary Approaches to Stop Hypertension (DASH) could greatly reduce the risk of postoperative infection [18]. Other preventative methods highlighted by the Society of Thoracic Surgeons Blood Conservation Guideline Task Force (2007) pertain to reductions in blood transfusions. One example is the use of drugs either preoperatively, such as erythropoietin to increase blood volume, or postoperatively, such as antifibrinolytics to reduce bleeding [18]. The incidence of postoperative infection is associated with heart failure in the literature [18], which aligns with our findings. The overall rate of postoperative infection in this study was quite high (32.7% of all patients in the sample), and this finding is concurrent with a local study that showed a 26.5% overall rate of postoperative infection [16]. Reducing the incidence of postoperative infections starts outside of the hospital setting, and we must make it a priority to promote healthier lifestyles, especially in populations where regular exercise and healthy habits are scarce.

The study has several limitations that should be taken into account. Firstly, this was a retrospective study with a single-center design and its generalizability was affected due to the small sample size. Given its retrospective and observational nature, the study had an inherent selection bias. Additionally, our study did not show any statistically significant association between blood transfusions and postoperative infections.

## Conclusions

Based on a thorough analysis of 364 patients undergoing a variety of cardiac surgical operations, including a multivariate analysis accounting for preoperative factors, we found a significant association between infections and hypertension, diabetes, increased preoperative activated partial thromboplastin time, and increased HbA1c. For every unit increase of red blood cells administered, there was a 9% increased risk of getting an infection. Type of operation, BMI, and duration of surgery were not associated with postoperative infections. However, females undergoing pure valvular surgery had a significant reduction in the rate of infection. As our center has a liberal transfusion protocol, more than 85% of patients received red blood cell transfusions, and the sample of patients without transfusions is too small to have statistical significance. Further studies with larger sample sizes and a multicenter prospective study design must be conducted to assess other risk factors as well as to gain deeper insights into the topic.
